# Vitamin D Deficiency During Pregnancy and Autism Spectrum Disorders Development

**DOI:** 10.3389/fpsyt.2019.00987

**Published:** 2020-01-31

**Authors:** Nicola Principi, Susanna Esposito

**Affiliations:** ^1^ Università degli Studi di Milano, Milan, Italy; ^2^ Pediatric Clinic, Pietro Barilla Children's Hospital, Department of Medicine and Surgery, University of Parma, Parma, Italy

**Keywords:** autism spectrum disorder, pediatric neurology, pregnancy, vitamin D deficiency, neurodevelopmental disorder

## Abstract

Autism spectrum disorder is a neurodevelopmental disorder characterized by reduced social interactions, impaired communications, and stereotypic and repetitive behavior with different degrees of severity. The etiology of autism spectrum disorder is unknown, although the interaction of genetic and environmental factors is believed to play a fundamental role in the process. The main aim of this narrative review is to discuss the current knowledge about the interrelationships between vitamin D deficiency during pregnancy and autism spectrum disorder development. Literature analysis showed that vitamin D supplementation during pregnancy plays a role in conditioning the development and function of the nervous system. Studies carried out *in vitro* and in experimental animals have shown that vitamin D deficiency can be associated with structural and functional abnormalities of the nervous system that can be observed in autism spectrum disorder patients. Moreover, it has been reported that vitamin D deficiency during pregnancy could be a risk factor for autism spectrum disorder development in the offspring, that children with autism spectrum disorder have significantly lower serum levels of vitamin D than normal children and that supplementation of vitamin D in autism spectrum disorder children is associated with a reduction in psychiatric manifestations. However, the data currently available do not adequately support the hypothesis that vitamin D may be a factor which contribute to the etiology of autism spectrum disorder. The effects of vitamin D supplementation during pregnancy should be better studied to establish whether and when fetal vulnerability is highest and if vitamin D supplementation is able to reduce the risk of structural and functional alterations of the nervous system and autism spectrum disorder development. The role of vitamin D after birth must be better defined to evaluate if vitamin D administration is potentially effective in reducing autism spectrum disorder manifestations.

## Introduction

For many years, it was thought that the role of vitamin D (VD) was to regulate calcium and phosphorus metabolism, thus assuring adequate bone mineralization and bone mass acquisition. In recent years, however, VD was shown to have significant extraskeletal activity in addition to its classically recognized actions. Adequate intake of VD appears to be essential to global physical and mental health, as suggested by the evidence that VD deficiency can be associated with several diseases, such as infections; asthma; inflammatory bowel diseases; obesity; metabolic syndrome; and neuropsychiatric manifestations, including autism spectrum disorder (ASD) (1–3).

ASD is a neurodevelopmental disorder characterized by reduced social interactions, impaired communications, and stereotypic and repetitive behavior with different degrees of severity (4). The etiology of ASD is unknown, although the interaction of genetic and environmental factors is believed to play a fundamental role in the process (5). In patients with ASD, a great number of variants in genes involved in brain development have been identified (6). Moreover, ASD is a familial disorder, and the concordance rate is significantly higher in monozygotic twins (50%) than in dizygotic twins (20%) (7). However, the heritability of ASD is no more than 50%, and in a relevant number of ASD cases, *de novo* structural genetic variations can be demonstrated (8). These findings suggest a multifactorial etiologic model in which external factors play a role in modulating the final structure and functions of the brain (8). Mercury intoxication, gestational infections, drug consumption during pregnancy, vaccine immunization, and VD have been under consideration as potential triggers (9). Studies carried out *in vitro* and in experimental animals have shown that VD deficiency is associated with a number of structural and functional abnormalities of the nervous system that can be observed in ASD patients (10). Moreover, it has been reported that VD deficiency during pregnancy could be a risk factor for ASD development in the offspring (11), that children with ASD have significantly lower serum levels of VD than in normal children (12) and that supplementation of VD in ASD children is associated with a reduction in psychiatric manifestations (13). However, definitive conclusions on the relationships between VD deficiency and ASD development cannot be drawn. Most of the studies planned in this regard are conflicting or have relevant methodological problems leading to inconsistent outcomes (14, 15). The main aim of this narrative review is to discuss the current state of knowledge about the potential interrelationships between VD deficiency during pregnancy and ASD and to provide suggestions for future research in this regard. Relevant articles published after 2005, reporting completed studies by searching electronic databases including MEDLINE, EMBASE, PubMed, were considered. Keywords searched included “vitamin D” and “autism” or “autism spectrum disorders”, “central nervous system”, “psychiatric disorders”, and “brain development”. Moreover, manual searches of reference lists of any systematic reviews identified in the previous step were performed.

## Vitamin D Pathways

Vitamin D3 (VD_3_) derives from sun exposure through the cleavage of the B ring of 7-dehydrocholesterol in the skin by ultraviolet B (UVB) radiation. This precursor molecule is initially hydroxylated in the liver by several cytochrome P450 (CYP) isoforms. Among these, CYP2R1 is thought to be the high-affinity 25-hydroxylase that produces 25-hydroxy-vitamin D_3_ (25(OH)D_3_), which is the metabolite that is routinely measured when VD status is assessed (16). 25(OH)D_3_ is further converted by the enzyme CYP27B1 into 1,25(OH)_2_D_3_, which is the active form of VD. Conversion occurs mainly in the kidney, although it has been demonstrated in other organs such as the brain. Finally, as excessive 1,25(OH)_2_D_3_ levels lead to absorptive hypercalcemia and/or hypercalciuria (17), the final concentrations of the active metabolite are regulated by CYP24A1 that occurs in the same tissues where active VD is synthetized, is upregulated in the presence of high 1,25(OH)_2_D_3_ concentrations and maintains 1,25(OH)_2_D_3_ at adequate, safe levels (18).

To initiate its action, the active form of VD must bind to VD receptor (VDR), a nuclear receptor and ligand-activated transcription factor that is a member of the superfamily of nuclear hormone receptors (19). Only after binding can the activated complex exert its genomic and nongenomic effects. VDR has been detected in many tissues and is present in many cells of the immune system. This would explain the influence of VD on the structure and function of several body organs and systems, including the nervous system (20).

## Serum Concentration of Vitamin D

Ideal values of VD serum levels are not precisely defined. In 2011, the Institute of Medicine, established that serum 25(OH)D_3_ concentrations of at least 20 ng/mL, corresponding to a recommended VD_3_ dietary allowance of 600 IU/day for ages 1 to 70 years and 800 IU/day for ages 71 years and older, could be considered adequate (21). This is because lower levels have been found to be associated with an increased risk of bone metabolism alterations, falls, and myopathy (22–25). However, several scientific societies have established an ideal value of 30 ng/mL, and a number of experts have suggested that, in order to assure prevention of certain skeletal effects, serum 25(OH)D_3_ concentrations must be maintained between 40 and 60 ng/mL. Some authors define values between 20 and 30 ng/mL as insufficiency and values lower than 20 ng/mL as deficiency (26). Others consider deficiency a value ≤10 mg/mL and insufficiency a value between 11 and 20 ng/mL (27). Another poorly defined quantity is the maximum tolerable level, with some evidence seeming to indicate that concentrations higher than 100 ng/mL can be maintained without clinical problems (28), while others indicate that serum 25(OH)D_3_ concentrations higher than 60 ng/mL in adults could be associated with an increased risk of death (29).

## Factors That Suggest a Relationship Between Vitamin D and Brain Structure and Function

### Detection of Vitamin D Receptor in the Nervous System

Several studies have shown that VDR is broadly distributed in brain neurons, peripheral neurons, and non-neuronal brain cells of experimental animals of all ages as well as adult humans, although the highest concentrations are in those regions of the nervous system that are required for critical functions. For example, VDR expression was found to be elevated in the prefrontal cortex and hippocampus, regions strictly related to learning, memory, and executive control. Moreover, VDR was detected in regions rich in dopaminergic neurons, showing potential linkage between VD and dopaminergic neurotransmission. Finally, it was reported that VDR expression regulates the structure of the fully mature brain, as an increase in VDR was associated with a reduction in cellular proliferation and a related increase in programmed cellular elimination (30–34).

The detection of VDR in nervous tissues has been considered evidence that VD exerts a relevant role in modulating the structure and function of the nervous system during development and maturity. Nevertheless, while the results obtained in experimental animals strongly support this statement, data collected in humans are very scarce and not sufficient to allow firm conclusions. No detailed information is available regarding the brain region in which VDR is first expressed. Moreover, it is not precisely defined in which period of gestation low VDR expression can lead to the brain alterations that are common in ASD. Translation of the data collected in rats to humans is practically impossible, as their developmental neurobiological stages are quite different. Furthermore, to complicate interpretation of available data, there is evidence that, at least in some cases, the laboratory methods used to detect VDR are unreliable. Wang and Deluca (35) studied several commercial monoclonal and polyclonal antibodies against VDR and found that most of them were nonspecific. Proteins other than VDR were identified, and false-positive results were reported. The presence of VDR was overestimated, as shown by evidence that, using more specific and sensitive methods, the presence of VDR in skeletal, cardiac, and smooth muscle, previously demonstrated in most of the studies, was no longer detected.

### Vitamin D Deficiency in Animals Causes Brain Structural and Functional Alterations Similar to Those Found in Humans With Autism Spectrum Disorder

Animal studies have indicated that VD performs several functions in the brain (36, 37). VD is a strong differentiating agent for developing nervous system cells. It controls the number and characteristics of nervous system cells, decreasing the expression of genes that regulate cell proliferation, apoptosis, and neurite formation (38). VD modulates calcium metabolism through the downregulation of voltage-sensitive L-type calcium channels to protect neuronal cells against excessive calcium influx (39). VD blocks the uptake of reactive oxygen species and inhibits the production of nitric oxide, thus limiting the damage induced by reactive oxygen species to lipid membranes (40). Finally, VD modulates a number of neurotrophic factors that are essential for hippocampal neuronal development and maintenance and for primary cortical neurons, and it also induces an anti-inflammatory response through regulation of the immune system (41, 42).

Starting from these premises, it is not surprising that VD deficiency has been associated with a number of significant structural and functional brain alterations. These are very important in the early stages of nervous system development, when VD deficiency causes severe and permanent structural and functional lesions, whereas the alterations are generally milder and limited to functional brain abnormalities when they occur in adults (10). VD-deficient animals exhibit neuroanatomical changes, such as increased brain volume and lateral ventricle size, calcium signaling abnormalities in the brain, and mitochondrial dysfunction. Moreover, they have increased sensitivity to oxidative stress, altered dopamine and serotonin signaling leading to alterations in neurotransmission and, finally, a significantly abnormal immune response associated with an increased risk of chronic inflammatory conditions and the development of autoimmune diseases (10). Finally, the behavior of offspring born to VD-deficient animals is similar to that of autistic young children (43). Interestingly, the timing of VD deficiency may be important to see/not to see ASD relevant phenotypes in rodents, and supplementation may have an impact on mouse models of ASD (44–46).

Moreover, the association between VD deficiency and ASD development seems to be supported by the evidence that some of the brain abnormalities described in VD-deficient animals can also be found in humans suffering from ASD. In the first year of life, ASD children have brain volumes larger than those of age-matched controls (47, 48), with increased gray matter volume and enlarged lateral ventricles (49, 50). Mutations of genes encoding calcium channels and modulating neuronal function have been found in patients with ASD (51). The prevalence of mitochondrial dysfunction (52) and the blood concentrations of reactive oxygen species (53) are higher in children with ASD than in those without. Dopamine signaling is sometimes, albeit rarely, altered in patients with ASD (54). Additionally, serotonin receptor concentration (55) and serotonin synthesis capacity (56) have been found to be reduced in adults with ASD.

Attractive though they may be, however, similarities between animals and humans are not sufficient to demonstrate that the structurally and functionally significant brain lesions observed in ASD are strictly related to VD deficiency. Other factors frequently found in ASD individuals can cause similar problems. For example, it has been demonstrated that most ASD children suffer from vitamin A deficiency (57), that this condition is associated with an exacerbation of psychiatric manifestations (58) and that supplementation of VD in ASD patients improves their condition (59) due to the role played by this vitamin in differentiation, proliferation, and development of the central nervous system. In addition, other environmental factors (nutritional or toxic) should be taken into account as risk factors of brain development (60).

### Maternal Vitamin D Deficiency and Impact on the Offspring

Maternal VD deficiency has been associated with an increased risk of ASD development in infants. The evidence for an elevated prevalence of ASD among children born to mothers with a putative reduction in VD skin synthesis during pregnancy has classically been considered evidence that hypovitaminosis D and ASD development are strictly related (61, 62). Babies conceived in winter months and those born to dark-skinned women who had immigrated to high-latitude countries were more frequently diagnosed with ASD than those conceived in summer months and those born to light-skinned migrants, respectively (61, 62). However, the results of these studies remain inconclusive for several reasons and recent studies did not seem to associate season of birth and ASD (63, 64).

Similar epidemiological studies did not confirm that the birth-date distribution of individuals with ASD was different from that of the general population (65). Studies on the relationship between ASD and sun exposure did not consider the possibility that genetics can significantly influence VD synthesis at the same dose of sun exposure. Moreover, the contribution of other factors essential for proper central nervous system development, such as the associated reduction in folate in its native state, has never been studied (66). Finally, as reported by Lee et al. (67), it was not calculated that indications of seasonality may be sensitive to the selection of time cut points; therefore, an arbitrary binning of time can lead to incorrect conclusions, masking existing trends or falsely indicating the presence of a trend.

Other studies with highly debatable conclusions include the studies that have tried to demonstrate a link between the VD status of pregnant women and the neuropsychiatric development of their infants. Magnusson et al. (68), using a register-based total population study (N = 509,639), studied the association between ASD with and without intellectual disability and lifetime diagnoses of maternal VD deficiency. The offspring's risk of ASD with, but not without, intellectual disability was found to be associated with reduced VD concentrations (adjusted odds ratio [OR) 2.51, 95% confidence interval [CI) 1.22–5.16, and OR 1.28, 95% CI 0.68–2.42). Chen et al. (69) enrolled 68 children aged 3 to 7 years with ASD and 68 typically developing children matched for age and gender. Measurement of maternal serum VD levels during the first trimester of pregnancy through the evaluation of archived maternal blood samples revealed that the prevalence of low VD status was more common among mothers of ASD patients in the VD group than among those with normal children (55.9% [95% CI 44.1%–67.7%) and 29.4% [95% CI 18.6%–40.2%), respectively). Moreover, mean serum VD levels were significantly lower in mothers of children with ASD than in controls (19.2 ng/mL [interquartile range (IQR): 15.8–22.9 ng/mL] vs. 24.3 ng/mL [IQR: 19.3–27.3 ng/mL; *p* < 0.001]). However, the trends in ASD prevalence in USA are not related to trends in serum 25(OH)D3 level, which are little changed compared to changes in ASD prevalence (70).

Finally, the degree of VD deficiency was positively associated with ASD severity as defined by scores on the Childhood Autism Rating Scale (CARS). Wu et al. (71) examined the associations between neonatal vitamin D status, the expression of maternal VD deficiency, and the risk of ASD development in the first three years after birth. A total of 310 ASD cases and 1,240 controls were studied. The median serum 25(OH)D_3_ level was significantly lower in children with ASD than in controls (7.0 ng/mL, IQR 4.96–10.9 vs. 16.0 ng/mL, IQR 11.0–19.5; *p* < 0.0001). The relative risk of ASD development was significantly higher in neonates in the lowest quartile (3.6, 95% CI 1.8–7.2; *p* < 0.001) than in those in the second and third quartiles (2.5, 95% CI 1.4–3.5; p = 0.024; and 1.9; 95% CI 1.1–3.3; p = 0.08). However, the results of these and similar studies must be interpreted with caution. In general, the associations were based on low numbers. Moreover, the samples were geographically limited, potentially reducing the generalizability of the results. VD status was derived from secondary-care records, and the appropriateness of the statistical methods used in the analysis was debatable. Finally, confounding factors such as drug consumption, psychiatric disorders, immigration, and disadvantaged social status were not analyzed. Causality cannot be inferred in such studies. A number of limitations were also present in the study by Vinkhuyzen et al. (72), although several attempts to avoid bias were made. These authors studied the association between gestational VD deficiency and a continuous measure of autism-related traits at ~6 years (the Social Responsiveness Scale, or SRS) in a large population-based cohort of mothers and their children (n = 4,229). VD levels were assessed from maternal mid-gestation sera and from neonatal sera. Elevated scores on the SRS were found in children with VD deficiency at both time points, whereas low VD at only one time point was not associated with abnormal SRS scores, suggesting that in order to be effective as a cause of ASD, VD deficiency must be persistent at least from mid-gestation until birth (72). However, some of the methodological problems previously reported were not completely solved. Moreover, as highlighted by the authors themselves, although the SRS is well suited to assess autism-related traits, poor communication can also be associated with a range of other childhood disorders. Findings based on epidemiology are susceptible to residual confounding, and it is feasible that VD status could serve as a proxy marker for other (unobserved) risk-modifying factors linked to SRS scores.

### Vitamin D Deficiency in Children

Multiple studies have reported inadequate VD status in children with ASD. Reduced intake and decreased exposure to UVB radiation have been considered as potential causes, but abnormal VD activity due to genetic polymorphisms of the genes encoding for the enzymes that hydroxylate VD_3_ or in the gene that encodes VDR was found to be the most common reason (73–75). A 2016 meta-analysis of 11 studies, including 870 ASD children and 782 typically developing children, demonstrated significantly lower serum 25(OH)D_3_ concentrations in ASD cases than in controls (12). This finding was reported also in more recent studies (76) and was considered a marker of ASD supporting the hypothesis that reduced availability of VD could increase the risk of ASD. Nevertheless, not all studies on this topic reported VD deficiency in ASD children. For example, Molloy et al. (77) compared plasma 25(OH)D_3_ concentrations in male ASD children 4 to 8 years old with or without dietary restrictions and in a group of typically developed age- and sex-matched subjects and found that most of the children had a very low VD concentration (< 20 ng/mL), without any difference among groups (p = 0.4). Uğur and Gürkan (78) investigated the serum levels of VD, calcium (Ca), phosphorus (P), alkaline phosphatase (ALP), and folate in 54 children aged 3 to 8 years with ASD and in 54 age- and gender-matched normal controls. VD, Ca, P, ALP, and folate levels of children with ASD were not different from those of the control group. Moreover, measures of psychiatric characteristics and level of cognitive development were not associated with laboratory findings. Finally, Basheer et al. (79) enrolled 40 children with ASD and 30 typically developing children aged 3 to 12 years and found that 93% of subjects in both groups had VD deficiency (25(OH)D_3_ < 12 ng/mL) or insufficiency (25(OH)D_3_ 12–20 ng/mL). However, VD status did not differ between groups, and no correlation between serum 25(OH)D_3_ levels and severity of ASD was found.

## Impact of Vitamin D Administration on Symptoms of Autism Spectrum Disorders

### Prophylaxis

The impact of VD administration during pregnancy on the mother and the conceptus before and after birth has been repeatedly studied. However, most studies on the topic enrolled very few subjects and were of low quality. A systematic review and meta-analysis of randomized controlled trials and of registered but unpublished trials carried out before September 2017 concluded that the evidence to date seems insufficient to draw conclusions. It was found that VD prophylaxis was not associated with benefits for the mother. Regarding the child, mean birth weight was slightly increased, and the risk of small-for-gestational-age birth was reduced. Those findings, however, were not robust in sensitivity or subgroup analyses (80).

A study specifically planned to evaluate the impact of gestational VD prophylaxis on the risk of ASD development in the child was carried out by Stubbs et al. (81). A dose of 5,000 IU/day was prescribed to pregnant women who already had a child with ASD, and children were given 1,000 IU/day from birth to their third birthday. ASD was diagnosed in only 1 out of the 19 children born to supplemented women, a significantly lower value than expected, as the prevalence of new ASD cases among children born to the mothers of previous children with ASD was found to be 20% (82). However, this finding must be considered with caution because the study was uncontrolled and included a very low number of pregnant women. Moreover, among enrolled individuals, the duration of VD administration varied, as approximately one-third of the cases were already receiving VD before becoming pregnant, whereas the others were prescribed VD in the first, second, and early third trimesters.

In conclusion, the data currently available do not support the hypothesis that VD prophylaxis during pregnancy impact on the risk of ASD development.

### Therapy

Only few studies have evaluated the role of VD supplementation for ASD treatment. Moreover, two of those that have reported positive results (83, 84) have been largely debated for the methods used to collect data and to evaluate results (85, 86). Furthermore, the most recent research has been retracted because a re-analysis of the data collected with the study discovered previously unidentified problems with missing data and recording irregularities that led the Editors to have no longer confidence in the findings reported (87). Consequently, these studies do not offer any reliable information and highlight how much readers must be vigilant when they try to draw firm conclusions from published studies. The first report of the use of VD_3_ for ASD treatment dates to 2014, when VD_3_ (150,000 IU intramuscularly every month and 400 IU/day orally for 2 months) was administered to a 32-month-old boy with ASD and VD deficiency. The results were impressive, as the child showed a significant improvement in ASD core symptoms in a very short time (88). Starting from this evidence, a few clinical trials specifically planned to evaluate the true value of VD_3_ in ASD therapy were performed. All those studies revealed that VD3 administration, regardless of the dosage used, was well tolerated and safe. Unfortunately, the results regarding effectiveness were quite different, and no definitive conclusion can be drawn at present.

Some examples can support this conclusion. Feng et al. (89) with an open-label study enrolling 215 children aged 3 to 11 years who had ASD and 285 healthy controls. Among ASD patients, 37 received VD_3_ (150,000 IU intramuscularly every month and 400 IU/day orally for 3 months). ASD symptoms were assessed using the Aberrant Behavior Checklist (ABC) and CARS scores. Serum levels of 25(OH)D_3_ were found to be significantly lower in ASD children than in healthy controls. Moreover, they were negatively correlated with total scores and language subscale scores on the ABC. Supplementation was effective, as both ABC and CARS scores were significantly reduced at the end of the study.

Moradi et al. (90) investigated the effect of perceptual-motor exercises, music, and VD consumption alone or in combination on the nerve growth factor (NGF) in children with high-functioning ASD. Patients receiving VD (50,000 IU per week for eight weeks) alone or in combination with perceptual-motor activities had the highest improvement in the levels of NGF, suggesting that supplementation of VD may be useful to improve health status in children with ASD.

On the contrary, VD_3_ supplementation was reported to have no effect in a study by Kerley et al. (91). These authors conducted a double-blind, randomized, placebo-controlled trial in which 38 children (mean age, 7.1 years) were enrolled. Among them, 18 were given VD_3_ (2,000 IU/day for 20 weeks) and 20 received placebo. Serum 25(OH)D_3_ levels were measured before VD_3_ administration and at the end of the study period. Before and after supplementation, subjective autistic measures rated by the parents and clinicians were assessed through the ABC, the SRS, and the Developmental Disabilities-Children's Global Assessment Scale (DD-CGAS). Although treated children showed a significant increase in serum 25(OH)D_3_ concentrations (23.4 ng/mL vs. 34.4 ng/mL) in comparison to patients receiving placebo (20.7 ng/mL vs. 20.2 ng/mL) (p = 0.0016), no effect was observed on the SRS, the DD-CGAS, or any of the five subscales of the ABC. In contrast, there was a trend toward decreased inappropriate speech in the placebo group (p = 0.08). No effect of VD supplementation was also shown by Mazahery et al. (92), who evaluated the efficacy of VD alone (2,000 IU daily), that of omega-3 long chain polyunsaturated fatty acids (OM; 722 mg docosahexaenoic acid) or both in children with ASD (n  = 73 aged 2.5–8.0 years). Outcome Social Responsiveness Scale (SRS) and Sensory Processing Measure (SPM) were used to measure the impact of the therapy. Only children receiving OM had greater improvements. The methods used to perform the studies may explain the different results. Some studies were open-label studies, while others, in contrast, were double-blind, randomized, placebo-controlled trials. Moreover, subjects with different characteristics at enrolment and during the period of VD_3_ supplementation were enrolled. Sun exposure of the enrolled patients during the study period was not always considered, although this could lead to a significantly different total supplementation of VD_3_. The ASD parameters of patients at baseline were not always comparable to those of controls. The presence of VDR gene polymorphisms was not taken into account. Different VD_3_ dosages were used. Compliance was not always adequately measured.

All these findings seem to suggest that the inclusion of VD_3_ administration among the treatment opportunities for ASD is premature, and further studies are needed to better define which children can gain a real benefit from supplementation and which dosage has the greatest probability of success with the lowest risk of adverse events. In general, oral administration of VD_3_ is followed by an increase in serum 25(OH)D_3_ levels. However, this does not always occur. In the study by Kerley et al. (93), among the 18 ASD patients who received VD3, 16 had an increase in 25(OH)D_3_ levels, whereas 2 had a reduction or no change, respectively. Children with ASD can have a lower response to supplementation than subjects without ASD. Despite a longer period of VD3 supplementation (2,000 IU/day for 20 weeks in comparison to the same dosage for 15 weeks), children with ASD had a significantly lower increase in serum 25(OH)D_3_ levels than children with asthma (+10.4 ng/mL vs. + 18.0 ng/mL). Reduced intake with food, altered absorption, reduced transformation of VD_3_ into 25(OH)D_3_ and genetic factors may explain this finding. Children with ASD may have dietary limitations because of sensory aversions or restricted interests or because parents eliminate certain dietary proteins, such as those included in milk, in an attempt to treat the ASD symptoms (94).

Gastrointestinal symptoms accompanied by structural and functional alterations, including increased intestinal permeability, deficient enzymatic activity of disaccharidases, increased secretin-induced pancreatic-biliary secretion, and abnormal fecal microbiota, are common in individuals with ASD (95, 96). Liver disease or genetic variations in enzymes that hydroxylate VD_3_ can lead to reduced serum 25(OH)D_3_ concentrations (97). Variants near genes involved in cholesterol synthesis, hydroxylation, and VD transport affect VD status (98). Genetic polymorphisms of VD-binding protein are associated with low 25(OH)D_3_ concentrations (99). The importance of these factors can be different from patient to patient, which means that, before VD supplementation is recommended, the characteristics of each patient should be ascertained.

## Conclusions

The data currently available do not adequately support the hypothesis that VD may be a factor which contribute to the etiology of ASD. The effects of VD deficiency and supplementation during pregnancy should be better studied to establish when fetal vulnerability is highest and if VD is able to reduce the risk of brain lesions and ASD development. The role of VD after birth must be better defined to evaluate if VD administration is potentially effective in limiting or reducing ASD manifestations.

## Author Contributions

NP and SE co-wrote the manuscript and both approved the final version.

## Conflict of Interest

The authors declare that the review was conducted in the absence of any commercial or financial relationships that could be construed as a potential conflict of interest.
